# Individual Differences in Cognitive Functioning Predict Compliance With Restoration Skills Training but Not With a Brief Conventional Mindfulness Course

**DOI:** 10.3389/fpsyg.2022.715411

**Published:** 2022-03-03

**Authors:** Freddie Lymeus

**Affiliations:** ^1^Institute for Housing and Urban Research, Uppsala University, Uppsala, Sweden; ^2^Department of Psychology, Uppsala University, Uppsala, Sweden

**Keywords:** mindfulness, effortless attention, compliance, cognitive functioning, restorative environments, nature, moderation, mediation

## Abstract

Mindfulness training is often promoted as a method to train cognitive functions and has shown such effects in previous studies. However, many conventional mindfulness exercises for beginners require cognitive effort, which may be prohibitive for some, particularly for people who have more pronounced cognitive problems to begin with. An alternative mindfulness-based approach, called restoration skills training (ReST), draws on a restorative natural practice setting to help regulate attention effortlessly and promote meditative states during exercises. Previous research has shown that a 5-week ReST course requires less effort and is attended by higher compliance with practice recommendations than a conventional mindfulness course, without compromising long-term outcomes. Here, we compare ReST and a formally matched conventional mindfulness course regarding the role that initial individual differences in cognitive functioning play in determining practice compliance and long-term improvements in dispositional mindfulness and cognitive functioning. In line with expectations, ReST participants who had more pronounced cognitive problems to begin with practiced more during the course, which in turn explained much of their improvement in dispositional mindfulness and cognitive functioning. In contrast, initial cognitive functioning did not explain practice and improvement in the conventional mindfulness course. The results provide further support for the potential utility of ReST as a low-effort method for enhancing cognitive functioning among people who would struggle with the demands of conventional mindfulness training. With careful integration of mindfulness practices with a restorative natural setting, these people can develop mindfulness and self-regulation capabilities without relying on effortful training.

## Introduction

### Attention Resource Dynamics in Conventional Mindfulness Training

Mindfulness training can improve multiple aspects of psychological functioning (Khoury et al., 2015; Sedlmeier et al., 2018). Not least, the possibility that mindfulness can enhance a person’s cognitive functioning in daily life attracts much interest. Recent meta-analyses (Cásedas et al., 2019; Sumantry and Stewart, 2021) affirm that mindfulness training strengthens several attention-related capabilities, including alerting, inhibition, shifting, updating, executive control, and working memory. Such improvements could feasibly improve general adaptation by reducing thought intrusions, distractions and resulting lapses and mistakes, and by enhancing performance in challenging tasks and bolstering resilience in stressful conditions.

Cognitive improvements with mindfulness training have often been explained with a training rationale, drawing on analogies with physical exercise and invoking notions of neural plasticity (Kabat-Zinn, 1990; Segal et al., 2002; Lutz et al., 2008; Malinowski, 2013; Fox et al., 2014, 2016; also see Tang and Posner, 2009; Bruya and Tang, 2018): Presumably, the type of meditation exercises that dominate in common mindfulness courses for beginners—so-called focused-attention exercises in which participants try to sustain attention to a given target such as the breath and repeatedly redirect attention when they get distracted—stimulate enhancements in the engaged attentional brain networks. Accordingly, mindfulness teachers consider that “systematic and intensive engagement in formal and informal mindfulness meditation practices is foundational” in mindfulness-based interventions (Crane et al., 2017, p. 994). Before they acquire a certain skill level, however, beginning meditators often struggle to maintain focus and perceive the practice as effortful (e.g., Hasenkamp et al., 2012; Lutz et al., 2015; Frewen et al., 2016).

Transient effort can be considered as a harmless part of the process of learning meditation, but can nonetheless be prohibitive for some (Baer et al., 2019): When people have low cognitive resources, willfully focusing attention is generally associated with aversive boredom and restlessness, and motivation to switch to more immediately rewarding activity (Hockey, 1997; Sarter et al., 2006; Inzlicht et al., 2018). Similar logic applies in health interventions, where the acceptability of a treatment diminishes when participants perceive the demands of participation as high relative to the level of resources they have available to invest (Sekhon et al., 2017). Even with interventions that credibly could confer important benefits, compliance failures may result, such as failure to meet recommendations for regular practice. Individual-level factors (i.e., the resources a person can invest) and program-level factors (i.e., the investment that a given treatment requires) thus interact to influence compliance. As per the training rationale, low practice compliance should confer less benefit. Some meta-analytic reviews indicate that the amount of completed mindfulness practice is associated with improvement across broad categories of cognitive outcomes and other aspects of health (Khoury et al., 2015; Sedlmeier et al., 2018; also see Lekkas et al., 2021 for a more nuanced analysis).

Several reviews of mindfulness research practices have called for more studies that account for undesired effects (which effort can be for some) and compliance problems (Davidson and Kaszniak, 2015; Nam and Toneatto, 2016; Baer et al., 2019). Some have specifically encouraged studies that connect individual- and program-level factors to determine which types of training suit different groups (Van Dam et al., 2018; Tang and Braver, 2020). Only a few studies have sought to explain compliance based on initial cognitive functioning: using different operationalizations (i.e., more thought intrusions and lower selective attention performance), Crane and Williams (2010), Lymeus et al. (2017), and Banerjee et al. (2018) indicate that participants who had more pronounced cognitive problems before the training subsequently engaged less with the course, practiced less, and dropped out more. In these studies, emotional problems apparently mattered little for compliance, supporting the idea that cognitive aspects are particularly and specifically relevant.

To summarize, the approach that is conventionally used in secular mindfulness training programs is effective on average but requires cognitive effort that can be prohibitive for many of those who have most to gain from learning mindfulness skills; that is, for people with more pronounced cognitive problems. Could this group be better served by a training approach that draws on environmental support to regulate attention during exercises?

### The ReST Approach to Mindfulness Training

Restoration skills training (ReST) is a mindfulness-based 5-week course set in a garden environment rich in natural features (see Lymeus, 2019; Lymeus et al., 2020). We developed ReST with the aims that it should be a less demanding yet at least similarly effective introduction to mindfulness training as a conventional mindfulness course. To accomplish that, ReST emphasizes open-monitoring practice, in which participants observe the stream of ongoing experience with minimal effortful to control it. Open-monitoring is presumably less effortful than focused-attention practice (Lutz et al., 2015; Tang et al., 2015; Fox et al., 2016). However, tradition and contemporary reasoning hold that beginners should train their cognitive capabilities in focused-attention exercises before transitioning to open-monitoring, because untrained beginners easily get distracted during open-monitoring.

As an alternative route to overcoming this lack, ReST combines open-monitoring with sensory exploration of the practice setting, where natural features and processes softly draw and hold attention in a bottom-up fashion. ReST thus integrates mindfulness theory and practices with knowledge of how nature experience can engage complementary processes effortlessly, improve access to attention-regulation capabilities, and counter stress (Kaplan, 2001; Tang and Posner, 2009; Lymeus, 2019). This is founded in research on restorative environments.

### Restorative Environments Research

Restorative environments research builds on the common observation that features in natural environments often draw and hold attention in an effortless and pleasant way (Ulrich, 1983; Kaplan and Kaplan, 1989; Kaplan, 1995; Hartig, 2021). Nature experience can thereby help regulate attention to present experience. Moreover, natural settings support an intuitive mode of processing and behaving, reduce routine mental contents and intrusive thoughts, and promote positive emotions that help counter stress (Bratman et al., 2015; Joye et al., 2016; Williams et al., 2018; Grassini et al., 2019). To investigate such phenomena, restorative environments studies normally assume or induce a state of mild stress or fatigue in participants, and then compare the outcomes of relatively brief (i.e., a few minutes to a few hours) resting activities in real or virtual nature versus built indoor or outdoor settings. Following nature experience, people on average improve more in performance on tests of attention control, cognitive flexibility, and working memory (see the meta-analytical review by Stevenson et al., 2018). This is presumably because they restore—or regain more complete access to—their cognitive capabilities. Concomitantly, nature experience increases positive affect and reduces psychophysiological stress (for reviews, see Hartig et al., 2014; McMahan and Estes, 2015; Twohig-Bennett and Jones, 2018).

Restoration is thus commonly thought to reinstate existing attention-regulation capabilities by returning a person from a state of depletion, through spontaneous and quick processes (Hartig, 2021). This contrasts with the training account that is commonly invoked to explain cognitive enhancements following meditation, where repeated exercise over time is thought to raise the base-level of attention-regulation capabilities.

The environmental approach to supporting attention regulation can help those who need it most without placing additional demands on their already decimated capacities (*cf*. Taylor et al., 1998; Hartig and Staats, 2006; Hartig, 2007; Wheeler et al., 2015; Twohig-Bennett and Jones, 2018; Bratman et al., 2019). On the other hand, the understanding is underdeveloped regarding how regular short-term restoration experiences can stimulate learning and lastingly improve psychological functioning (Dzhambov et al., 2019; Hartig, 2021). Mindfulness training offers a structured progression by which participants can gain such widely useful skills. ReST seeks to integrate these advantages.

### Previous Findings on ReST

In comparisons with conventional mindfulness training (CMT), Lymeus et al. (2018) used a restorative environments design with attention tests obtained directly before and after meditation practice and saw that ReST participants improved (i.e., restored attention performance, consistent with restful effortlessness in the practice), whereas CMT participants deteriorated (consistent with resource depletion in effortful practice). Lymeus et al. (2019) showed that higher perceived restorative quality in the meditation setting facilitated state mindfulness during the ReST classes and partially explained a higher participant retention rate in ReST compared to CMT. Furthermore, Lymeus et al. (2020) showed that the more effortless ReST approach to mindfulness training performed no worse than CMT in improving dispositional mindfulness, cognitive functioning in daily life, and chronic stress. ReST could thereby help a larger number of participants establish a regular meditation habit and enjoy its benefits. However, one of the questions that motivated the development of ReST remains unanswered: Does ReST particularly help the most vulnerable participants, who would presumably be least likely to complete more effortful training and who have most to gain from learning mindfulness skills?

### Aims of the Present Study

The underlying study (see Lymeus et al., 2019, 2020) involved four data collection rounds with the same basic design, contrasting ReST and CMT. The two courses were closely matched in terms of structure and contents. Both involved weekly classes over 5 weeks, instructions to practice with given formal and informal meditation assignments on most days, and to keep daily records of the practice. Before and after the course, participants rated their cognitive functioning and dispositional mindfulness. Building on the reasoning outlined above, we formulated expectations regarding the associations between ReST and CMT participants’ initial cognitive functioning and subsequent compliance with the practice assignments, further assuming that more practice would be associated with better outcomes.

Specifically, we expected that among ReST participants, those who had poorer cognitive functioning to begin with would practice more (per the rationale that they should be more drawn to practices that support restoration) than participants who had better initial cognitive functioning. Among CMT participants, we expected that those who had poorer cognitive functioning to begin with would practice less (because they should be more averse to effortful practices). We thus posed a moderation hypothesis, where the association between initial cognitive functioning and practice is moderated by course type.

We also posed a serial mediation hypothesis. Per the rationale that practice trains mindfulness skills, which in turn generalize and improve cognitive functioning in daily life, we expected that participants who practiced more would improve more in dispositional mindfulness, which in turn would explain improvement in cognitive functioning.

**Figure 1:**
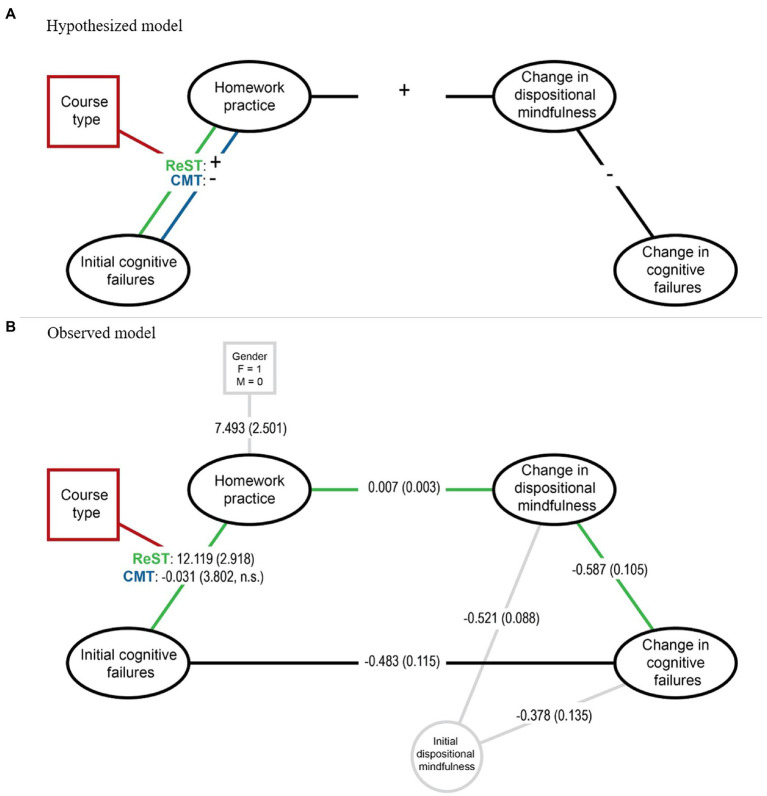
Panel **(A)** visualizes the hypothesized moderated mediation effect, where participants who have relatively poor initial cognitive functioning (i.e., higher scores on the Cognitive Failures Questionnaire) were expected to complete more of the assigned mindfulness exercises (Homework practice) if they had been randomly assigned to a 5-week restoration skills training (ReST) course, whereas poor initial cognitive functioning was expected to be negatively related to mindfulness practice for participants who were assigned to a formally matched conventional mindfulness training (CMT) course. With both courses, homework practice was expected to explain improvement in dispositional mindfulness (assessed with the Five Facet Mindfulness Questionnaire) which in turn would explain improvement in cognitive functioning. Panel **(B)** shows the observed coefficients for the hypothesized paths as well as those other effects that were observed to be significant, including the effects of two covariates (Gender and Initial dispositional mindfulness). The association between Initial cognitive functioning and Homework practice was as expected for ReST (*n* = 55) but non-significant and virtually null for CMT (*n* = 44). Homework practice and improved dispositional mindfulness mediated improvements in cognitive functioning in serial, as expected. Coefficients are unstandardized. Initial scores are mean item ratings: possible scores for the Cognitive Failures Questionnaire = 0–4 and for the Five Facet Mindfulness Questionnaire = 1–5. Change was calculated as the score after the course—score before the course. Homework practice is the total sum of completed formal and informal mindfulness exercises as measured with daily structured practice records.

These theoretically derived expectations were integrated in a conceptual model (see panel **(A)** of Figure 1). Note that the omitted lines mean that we posed no specific hypotheses regarding those associations, not that we necessarily expected null findings.

## Materials and Methods

This section is limited to methods of direct relevance here. For aspects that are peripheral to the present aims and analyses and that have been detailed elsewhere, we refer readers to the relevant publications.

### Design and Participants

The study was approved by the regional ethical review board (registration number: 2013/033). It was conducted in four data collection rounds between 2013 and 2017, three rounds taking place in spring and one in fall. In each round, we recruited participants from a university campus through flyers advertising a study about mindfulness training (without further specification). We targeted students because they have elevated levels of stress and other psychological health issues compared with the general population (e.g., Vaez and Laflamme, 2008; Christensson et al., 2011) and because their cognitively demanding academic pursuits, if successful, can contribute to shaping the future. Interested students with self-perceived concentration problems and stress and who passed screening for major health issues were stratified by gender and randomly assigned to ReST or CMT. They completed assessments directly before and after the 5-week course and kept structured daily records of completed mindfulness practice assignments.

The initial sample size was determined with a view to group differences in change in the primary outcomes and anticipated <25% dropout. Lymeus et al. (2020) detail recruitment procedures and provide a CONSORT diagram and Lymeus et al. (2019) analyze the dropout patterns. Of the total sample of 139 who started either ReST or CMT, 61 completed ReST and 52 completed CMT. However, some had missing homework or outcome data and were excluded from analyses. Thus, 55 ReST participants (64% females, median age = 24) and 44 CMT participants (68% females, median age = 24) were included in the analyses. Distributions were similar for gender (*χ*^2^[1] = 0.22, *p* = 0.636, *φ* = 0.048) and age (*U* = 1375.00, *p* = 0.243).

### Mindfulness Courses

The two courses ran in parallel and were closely matched in structure and homework requirements. Both entailed one 90-min class per week over 5 weeks and instructions to complete given 15–20-min formal and informal practice assignments on most days. The conceptual contents and practice rationales were also designed to mirror each other but used different formulations to align with the respective practice approach. Lymeus (2019) details the development, principles, contents, and settings of the mindfulness courses.

ReST was given in a botanic garden and used a practice approach based on open-monitoring and sensory exploration. Note that the ReST homework assignments encouraged but did not require practice in nature because this could have introduced undue constraints. Rather, they instructed participants to draw on sensory contacts with their environment wherever they practiced. CMT was given indoors in a campus building and built on the curriculum and practice principles of the established Mindfulness-Based Stress Reduction (MBSR; Kabat-Zinn, 1990), using mainly practices targeting bodily, emotional, and cognitive aspects of experience.

### Measures

The participants rated their cognitive functioning in the last month with the Cognitive Failures Questionnaire (CFQ; Broadbent et al., 1982). The CFQ was presented as an indicator of cognitive vulnerability to stress. It has 25 questions about how often a person made mistakes in the areas of perception, action, and memory and gives a higher score for poorer levels of cognitive functioning. Cronbach’s α before the course was 0.87 and after, 0.88.

The participants rated their dispositional mindfulness in the last month with the Five Facet Mindfulness Questionnaire (FFMQ; Baer et al., 2006). The FFMQ has 29 items about how often a person experienced non-judgment, non-reactivity, acting with awareness, observing, and describing and gives a higher score for higher dispositional mindfulness. α before the course was 0.85 and after, 0.87.

In the second, third, fourth, and fifth class, the participants handed in registration sheets where they had indicated when they completed the given formal and informal assignments during the preceding week. Eighteen missing homework reports (4.5% of the reports) were replaced with the participants’ value for the preceding week. Where two or more sequential reports were missing, they were not replaced and the participant was dropped from analyses (see “Design and Participants”).

### Statistical Analyses

We tested the moderated serial mediation model with conditional process analysis using the PROCESS macro for SPSS (model 83; Hayes, 2017). The initial CFQ score was entered as the individual average of all item responses. Change in CFQ and FFMQ was entered as change scores (after course−before course). Homework practice was entered as the total sum of registered practice. Several covariates were also considered: data collection round, age, gender, and initial FFMQ score (average item response). Only gender and initial FFMQ score contributed to explaining variance at different steps and were retained. The model held for including round and age but these did not improve the model and so were dropped.

Testing the model involved three linear regressions (steps). The first step predicted homework practice from initial CFQ score, course type, and initial CFQ score x course type. The second step predicted change in FFMQ from initial CFQ score and homework practice. The third step predicted change in CFQ from initial CFQ score, homework practice, and change in FFMQ. The two retained covariates were included in each step. Considering each of these analyses separately, the sample of *N* = 99 gave a test sensitivity of 0.84–0.87 given moderate expected effects and *α* = 0.05. Finally, the analyses used 10 k bootstrap samples to produce bias-corrected and heteroscedasticity consistent (HC3) 95% confidence intervals for the indirect effects and the index of moderated mediation.

## Results

Table 1 provides descriptive statistics for the variables and the bivariate correlations between them. ReST and CMT had similar initial values (CFQ, *t* = 0.88, *p* = 0.379; FFMQ, *t* = 0.69, *p* = 0.494) and homework practice rates (*t* = 0.57, *p* = 0.572).

**Table 1:** Means and standard deviations for all variables and bivariate correlations between them, separately for restoration skills training (ReST; *n* = 55) and conventional mindfulness training (CMT; *n* = 44) course completers.

		M (SD)	Initial CFQ	Initial FFMQ	CFQ change	FFMQ change	Homework practice

ReST	Initial CFQ	1.83 (0.50)	1				
	Initial FFMQ	3.03 (0.40)	−0.261	1			
	CFQ change	−0.27 (0.54)	−0.552^**^	0.121	1		
	FFMQ change	0.28 (0.43)	0.134	−0.484^**^	−0.455^**^	1	
	Homework practice	27.85 (13.21)	0.456^**^	−0.129	−0.439^**^	0.365^**^	1
CMT	Initial CFQ	1.74 (0.50)	1				
	Initial FFMQ	2.97 (0.50)	−0.646^**^	1			
	CFQ change	−0.23 (0.37)	−0.456^**^	0.269	1		
	FFMQ change	0.34 (0.45)	0.383^*^	−0.630^**^	−0.571^**^	1	
	Homework practice	29.27 (11.22)	−0.013	−0.015	−0.177	0.112	1

The variables are ratings of cognitive functioning (Cognitive Failures Questionnaire, CFQ; scale range 0–4 where higher values correspond to poorer cognitive functioning and negative change scores correspond to improvement), dispositional mindfulness (Five Facet Mindfulness Questionnaire, FFMQ; scale range 1–5 where higher values correspond to higher dispositional mindfulness and positive change scores correspond to improvement), and total completed formal and informal homework practice during the mindfulness course. * indicates *p* < 0.05 and ** indicates *p* < 0.01.

The model held up well at each step and the final regression explained much of the variation in change in CFQ (*R* = 0.71 [*R^2^* = 0.50], *p* < 0.001). Table 2 provides complete test statistics and coefficients for each step. Panel **(B)** of Figure 1 shows the observed coefficients for comparisons against the expectations visualized in panel **(A)**.

**Table 2:** Results from the successive steps in testing for the effect of initial cognitive functioning (Cognitive Failures Questionnaire) on improvement in cognitive functioning with the mindfulness training courses, as mediated in serial through the total number of completed homework exercises and improvement in dispositional mindfulness (Five Facet Mindfulness Questionnaire), and moderated in the first step by course type (restoration skills training [ReST; *n* = 55] or conventional mindfulness training [CMT; *n* = 44]).

Step 1		DV = Homework					
		***R (R^2^)***	***MSE***	***F(HC3)***	***p***	***df1***	***df2***
Model summary:		0.462 (0.213)	125.984	5.692	<0.001	5	93
		***Coefficient***	***SE***	***t***	***p***	***LLCI***	***ULCI***
Model specification:	Constant	18.797	12.738	1.476	0.143	−6.498	44.093
	Initial cognitive functioning	−0.031	3.802	−0.008	0.993	−7.581	7.518
	Course type	−23.398	9.143	−2.560	0.012	−41.554	−5.242
	Initial cognitive functioning x Course type	12.150	4.830	2.516	0.014	2.559	21.741
	Initial dispositional mindfulness	1.824	2.725	0.670	0.505	−3.586	7.235
	Gender	7.493	2.501	2.996	0.004	2.526	12.460
Conditional effects of Initial cognitive failures:	ReST	12.119	2.918	4.154	<0.001	6.325	17.913
	CMT	−0.031	3.802	0.008	0.993	−7.581	7.518
**Step 2**		**DV = Change in dispositional mindfulness**					
		***R (R^2^)***	***MSE***	***F(HC3)***	***p***	***df1***	***df2***
Model summary:		0.617(0.380)	0.124	17.899	<0.001	4	94
		***Coefficient***	***SE***	***t***	***p***	***LLCI***	***ULCI***
Model specification:	Constant	1.679	0.371	4.526	<0.001	0.943	2.416
	Initial cognitive functioning	−0.047	0.092	−0.512	0.610	−0.230	0.136
	Homework	0.007	0.003	2.403	0.018	0.001	0.012
	Initial dispositional mindfulness	−0.521	0.088	−5.950	<0.001	−0.695	−0.347
	Gender	0.130	0.086	1.506	0.135	−0.041	0.301
**Step 3**		**DV = Change in cognitive failures**					
		***R (R^2^)***	***MSE***	***F(HC3)***	***p***	***df1***	***df2***
Model summary:		0.706(0.498)	0.118	14.046	<0.001	5	93
		***Coefficient***	***SE***	***t***	***p***	***LLCI***	***ULCI***
Model specification:	Constant	2.025	0.557	3.636	<0.001	0.919	3.131
	Initial cognitive functioning	−0.483	0.115	−4.215	<0.001	−0.710	−0.255
	Homework	−0.005	0.003	−1.462	0.147	−0.011	0.002
	Change in dispositional mindfulness	−0.587	0.105	−5.600	<0.001	−0.795	−0.379
	Initial dispositional mindfulness	−0.378	0.135	−2.797	0.006	−0.646	−0.110
	Gender	0.046	0.076	0.605	0.547	−0.105	0.197

*Initial dispositional mindfulness and gender are included as covariates. Course type = ReST (1)* vs. *CMT (0). Gender = Female (1) vs. Male (0). CFQ scale = 0–4 where higher values correspond to poorer cognitive functioning and negative change scores correspond to improvement; FFMQ scale = 1–5 where higher values correspond to higher dispositional mindfulness and positive change scores correspond to improvement. Homework is the total sum of reported completed formal and informal homework exercises over the 5-week course. Coefficients are unstandardized. Standard errors (SE) are heteroscedasticity consistent (HC3). LLCI and ULCI are the lower and upper limits of 95% confidence intervals*.

As expected, the serial mediation path (*initial CFQ > homework practice > change in FFMQ > change in CFQ*) was moderated by course type: index of moderated mediation = −0.049 (CI_boot_: −0.111, −0.006). The indirect effects were, for ReST = −0.048 (CI_boot_: −0.104, −0.009) and for CMT = −0.000 (CI_boot_: −0.038, 0.031). Hence, the serial mediation path was in the expected direction for ReST but virtually null (in contrast to the expected opposite directionality compared to ReST) for CMT. The two shorter paths about which we had not formulated any particular expectations were non-significant: for the moderated path *initial CFQ > homework practice > change in CFQ*, ReST = −0.055 (CI_boot_: −0.146, 0.016) and CMT = 0.000 (CI_boot_: −0.036, 0.049) and for the unmoderated path *initial CFQ > change in FFMQ > change in CFQ* = 0.028 (CI_boot_: −0.067, 0.135).

**Figure 2:**
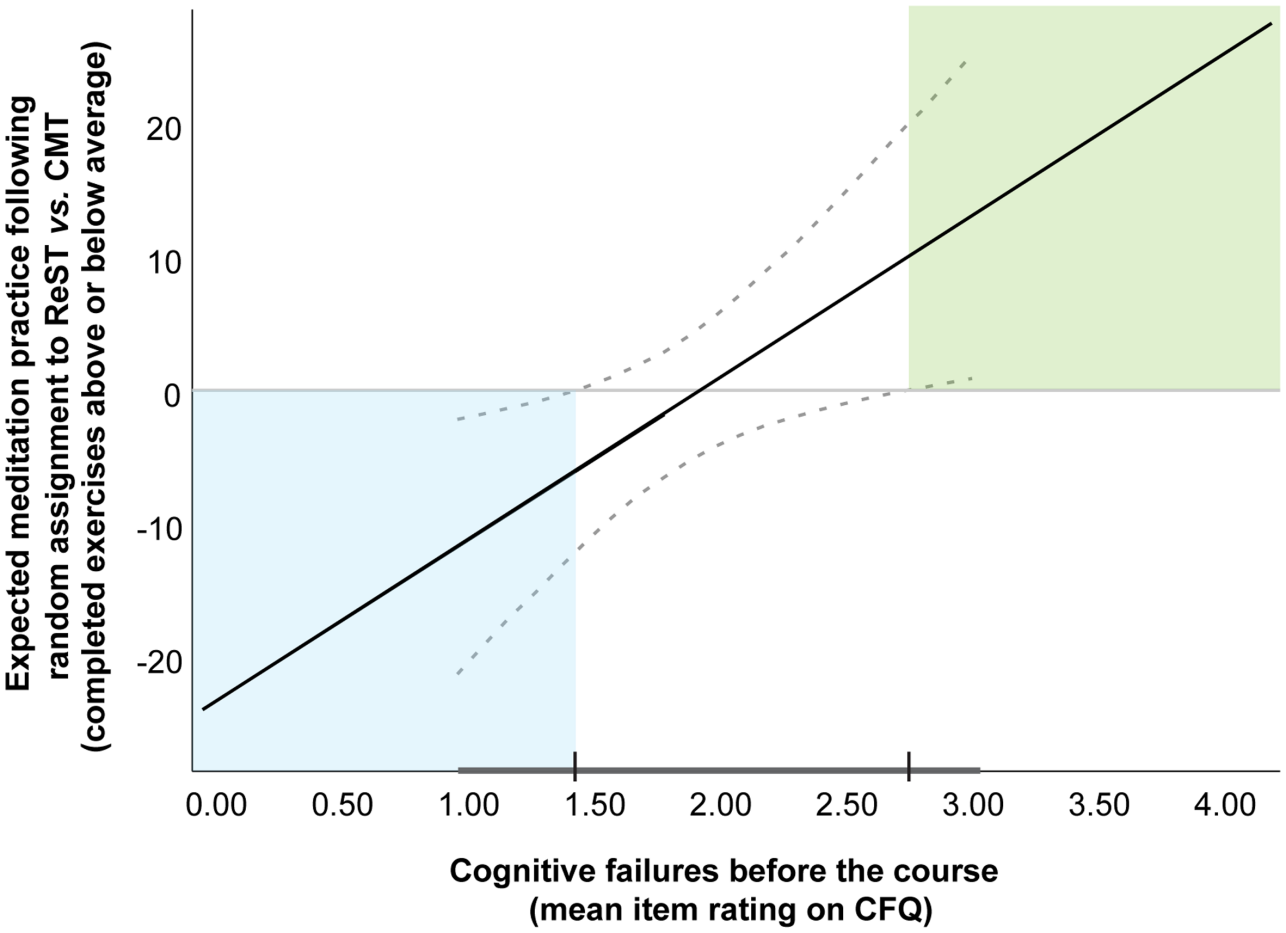
Johnoson–Neyman plot showing the observed moderation of the relationship between self-ratings of cognitive functioning before the course (average item response on the Cognitive Failures Questionnaire) and subsequent meditation practice (number of completed formal and informal mindfulness exercises above or below the sample average) by course type. Higher meditation scores indicate that participants with a given level of initial cognitive functioning completed more homework practice if they had been randomly assigned to restoration skills training (ReST) vs. a formally matched conventional mindfulness training (CMT) course. The dotted lines show the 95% confidence intervals. The thick line along the X-axis shows the range of observed values and the tick marks mark the significance regions: the levels of cognitive functioning above and below which the homework completion rates differed reliably between the courses.

Figure 2 provides a Johnson-Neyman plot of the moderated mediation effect at step 1, showing how initial CFQ score was associated with different practice rates in the two courses. The significance regions indicate that participants with an initial mean item rating on CFQ above 2.74 (on the scale of 0–4 where 2 = “occasionally” and 3 = “quite often” with regard to the occurrence of cognitive lapses) practiced reliably more if they had been randomly assigned to ReST rather than CMT. In contrast, participants with an initial mean item rating below 1.43 (where 1 = “very rarely”) practiced reliably less if they were assigned to ReST.

## Discussion

The results support the expectations for ReST: Participants who had poorer initial cognitive functioning practiced more if they were randomly assigned to ReST rather than CMT, which in turn predicted greater improvements in dispositional mindfulness and cognitive functioning. For CMT, however, the results were not as expected: The serial mediation path linking initial cognitive functioning, homework practice, and improvement was virtually null rather than the inverse of the effect observed for ReST. In other observations, females practiced more than males.

The direct paths between initial cognitive functioning and change in cognitive functioning, and between initial dispositional mindfulness and outcomes are difficult to interpret because unknown parts of those associations are likely statistical artifacts (e.g., regression to the mean). It is, however, essential to include initial values when modeling change (see Vickers and Altman, 2001; Clifton and Clifton, 2019).

The findings suggest that ReST preserves a core feature of restorative nature experience even when it is integrated with a mindfulness-based training approach: it particularly benefits those who need it most. This is congruent with previous findings that ReST practices permit and promote attention restoration during meditation (Lymeus et al., 2018) and that the high restorative quality of the garden setting used in this research supported ReST participants in achieving deeper meditative states and in maintaining compliance over the course duration (Lymeus et al., 2019).

Previous studies indicated that the conventional approach to mindfulness training may selectively drive out participants with more pronounced cognitive problems (Crane and Williams, 2010; Lymeus et al., 2017; Banerjee et al., 2018). That pattern could not be reaffirmed here. Even so, the present results support the higher suitability of ReST compared to the conventional approach for people with relatively more pronounced cognitive problems.

The Johnson-Neyman significance regions give confidence that ReST particularly supported participants with an initial mean item rating on CFQ above 2.74. Given the relatively wide CI and lack of extreme values in the sample, this is only a preliminary estimate of the level of cognitive functioning at which a person may be better served by ReST than CMT. The significance regions also indicate that the ReST approach might be less suited for people with very low levels of cognitive problems.

### Limitations and Needs for Further Research

Regarding the moderate sample, the study was sufficiently powered for the three regression analyses underlying the model. Bootstrap sampling for estimation of the moderated serial mediation paths allowed us to affirm a marked difference in the associations between initial cognitive functioning, practice, and course outcomes. While the virtual null association between initial cognitive functioning and practice in CMT makes it unlikely that a true effect was erroneously rejected, a larger sample could have bolstered confidence in the conclusions. Furthermore, the average initial level of cognitive functioning among the participants was moderate, as we recruited active and otherwise healthy university students. The observed variation in cognitive functioning covered much of the CFQ scale but not its extremes. Future studies must determine how ReST works for people with cognitive problems at clinical levels. Future studies can also target other groups that may struggle with CMT.

Regarding the measures, we relied on self-reports. Both CFQ and FFMQ are established measures but additional assessments of real-life performance and behavior could have bolstered confidence in the conclusions. For the practice records, daily registrations of well-defined and positively valued behaviors are generally quite reliable (Korotitsch and Nelson-Gray, 1999). Further analyses of temporal patterns and quality in the practice might have added nuance and predictive strength (Lekkas et al., 2021).

The study compared specific mechanisms behind improvements seen with two *bona fide* mindfulness courses, but the design does not allow conclusions about the relative contributions of the setting and other aspects of the practice approach in ReST. Like others researching connections between mindfulness and nature experience (e.g., Djernis et al., 2019; Macaulay et al., 2022), we consider that mindfulness training that draws on affordances in natural settings likely requires some adaptations of the practice approach, as reflected in ReST. In exercises that emphasize focused attention to internal aspects of experience, a rich natural setting will likely distract more than support beginning practitioners. This assumption is pending empirical evaluation; yet, we chose not to include a condition that we thought might be relatively unhelpful (i.e., CMT in the garden).

Furthermore, the CMT we used built on MBSR but used a briefer format. Brief formats are common (Van Dam et al., 2018) and often effective (Carmody and Baer, 2009). However, the null results for CMT did not align with expectations based on previous studies (Crane and Williams, 2010; Lymeus et al., 2017; Banerjee et al., 2018): Associations between initial cognitive functioning, compliance, and outcomes may be more pronounced in longer and more demanding conventional mindfulness courses.

Yet, other factors than the specific practice approach and setting may also influence compliance, including how different concepts and a rationale for regular practice are communicated. Both ReST and CMT included credible and attractive conceptual explanations and motivations to practice, although in terms that aligned with their respective practice approaches (see Lymeus, 2019). While the courses were matched in terms of the included contents, the different formulations of those contents may have contributed to compliance differences. The unexpected null result observed for CMT indicates that the course motivated and prepared participants to practice at moderate average levels even though the practice approach required effort. Hence, the CMT in this study was not bad for cognitively weaker participants. They were, however, better helped by ReST.

## Conclusion

This study provides further support for the utility of ReST as a low-effort method for enhancing cognitive functioning among people who would struggle with the demands of CMT. With careful integration of mindfulness practices and a restorative natural setting, these people can develop mindfulness and attention-regulation capabilities without relying on effortful training. More broadly, the study contributes to the developing understanding of the mechanisms behind outcomes in different forms of mindfulness training, and how they suit people with different needs based on individual differences. It also contributes to the growing literature on connections between nature experience and mindfulness.

## Data Availability Statement

The raw data supporting the conclusions of this article will be made available by the author, without undue reservation.

## Ethics Statement

The studies involving human participants were reviewed and approved by the Regional ethical review board for Uppsala (reference number 2013/033). The patients/participants provided their written informed consent to participate in this study.

## Author Contributions

FL designed the study, led the data collection, analyzed the data, and wrote the manuscript.

## Funding

This research was supported by the Department of Psychology and the Institute for Housing and Urban Research at Uppsala University. No particular grant or external funding was received.

## Conflict of Interest

The author declares that the research was conducted in the absence of any commercial or financial relationships that could be construed as a potential conflict of interest.

## Publisher’s Note

All claims expressed in this article are solely those of the authors and do not necessarily represent those of their affiliated organizations, or those of the publisher, the editors and the reviewers. Any product that may be evaluated in this article, or claim that may be made by its manufacturer, is not guaranteed or endorsed by the publisher.
